# Mitigating unintended consequences of co‐design in health care

**DOI:** 10.1111/hex.13308

**Published:** 2021-08-02

**Authors:** Éidín Ní Shé, Reema Harrison

**Affiliations:** ^1^ School of Population Health University of New South Wales Kensington NSW Australia; ^2^ Centre for Health Systems and Safety Research Australian Institute of Health Innovation Macquarie University Sydney NSW Australia

**Keywords:** co‐design, health‐care improvement, inclusive involvement, public and patient involvement, seldom heard, unintended consequences

## Abstract

**Background:**

Co‐design and associated terms are increasingly being used to facilitate values‐based approaches to health‐care improvement. It is messy and complex, involving diverse actors.

**Methods:**

We explore the notion that initiatives have outcomes other than initially planned is neither new nor novel but is overlooked when thinking about co‐design. We explore some of the unintended consequences and outline some optimal conditions that can mitigate challenges.

**Discussion:**

Although co‐design approaches are being applied in health care, questions remain regarding its ability to produce gains in health outcomes. Little is known about determining whether co‐design is the most suitable approach to achieve the given project goals, the levels of involvement required to realize the benefits of co‐design or the potential unintended consequences. There is a risk of further marginalizing or adding burden to under‐represented populations and/or over‐researched populations.

**Conclusion:**

Undertaking a co‐design approach without the optimal conditions for inclusive involvement by all may not result in an equal partnership or improve health or care quality outcomes. Co‐design requires on‐going reflective discussions and deliberative thinking to remove any power imbalances. However, without adequate resources, a focus on implementation and support from senior leaders, it is a tough ask to achieve.

**Patient or Public Contribution:**

This viewpoint article was written by two academics who have undertaken a significant amount of PPI and co‐design work with members of the public and patient's right across the health system. Our work guided the focus of this viewpoint as we reflected on our experiences.

## 1 INTRODUCTION

*Co‐design* (Box 1) and the associated terms of *co‐creation, experience‐based co‐design, co‐production, and public and patient involvement* are approaches to research inquiry increasingly used by clinicians and health service researchers to facilitate values‐based approaches to health‐care improvement. Values‐based approaches are those that seek to embed deeply held ideals that patients, families, health professionals and other stakeholders consider to be important in decision making about proposed improvements and their implementation.^1^ These approaches have been described as the new ‘*Zeitgeist*—the spirit of our times’^2^ and are advocated as the best approach for a ‘third era’ of medicine.^3^ Co‐design has attracted increasing interest in the context of shifting health‐care priorities towards user‐centric care and outcome measurement.^4^, ^5^, ^6^

Co‐design approaches are not new; with foundations in the humanities and social sciences, 'action research', 'emancipatory’ and ‘participatory’ approaches have focused on advancing transformation, empowerment of the person, social justice, and advocacy and translation since the early 1990s.^7^, ^8^ These approaches and terms may be new for many working and undertaking quality improvement and research in health systems, but their foundations are rooted in the humanities and social sciences. Their focus is on advancing transformation, empowerment of the person, social justice, and advocacy and translation.^8^, ^9^ When reviewing the literature, the separate reflections of Barnes and Oliver are worth revisiting.^7^, ^10^ They question whether emancipatory approaches are realistic if those leading it are interested in improving the broader structures for everyone involved.^11^, ^12^ We argue that these questions need to be reflected upon when undertaking co‐design and associated terms in health care.

Health systems internationally have shown a strong appetite for clinicians to demonstrate the use of co‐design approaches in implementing change‐focused interventions and service improvement.^2^, ^11^, ^12^ Health‐care agencies and systems have also identified co‐design as central to supporting patient‐centred care, illustrated in the increasing support for co‐design approaches in health research funding schemes worldwide.^13^, ^14^, ^15^

BOX 1 **What is co‐design?**

Co‐design is a values‐based methodology.^1^ Within healthcare the process includes bringing together service users, clinical and non‐clinical staff and, at times, relevant support and advocacy groups to work together to improve or refine elements of the care systems, services or processes. It is focused on the reality of healthcare contexts and of healthcare staff work environments.^7^ At its core is open reciprocal democratic dialogue where all participants contribute equally.^8^ This approach moves from consulting to enabling the involvement of all from the outset. It ensures and supports all relevant partners to be involved in defining the problem, designing the solution and monitoring and championing the implementation.

There is diverse and ever‐increasing evidence of widespread use of co‐design and associated approaches to improve health care and create change for quality improvement.^16^, ^17^, ^18^, ^19^, ^20^, ^21^ Successful co‐design has also been utilized in patient safety interventions,^22^ the development of frailty pathways,^19^ the development of telehealth services^23^ and within lean, a structured quality improvement approach in health care.^24^

## 2 UNINTENDED CONSEQUENCES FROM CO‐DESIGN

The notion that initiatives have outcomes other than initially planned is neither new nor novel but has been overlooked when thinking about co‐design.^25^ Although co‐design approaches are increasingly applied in health care, questions remain regarding its ability to produce gains in health outcomes.^26^, ^27^ Little is known from the literature on determining whether co‐design is the most suitable approach to achieve the given project goals, the levels of involvement required to realize the benefits of co‐design or the potential unintended consequences.^16^, ^28^, ^29^, ^30^, ^31^ Can we potentially do more harm than good?

The application of co‐design and its value for improving long‐term health‐care outcomes—including the depth and duration of resultant change—has been contested.^16^, ^32^, ^33^ Recent reviews of the literature found the involvement of service users/patients still at low and often tokenistic levels.^16^, ^26^ Apparent gaps in scientific rigour, tensions and hierarchical power differences between those involved in the process, coupled with a focus on short‐term outcomes, raise questions regarding the use of co‐design.^16^, ^34^ Few works provide any evidence or guidance on supporting the involvement of health‐care staff who may have a diverse range of needs to support their full participation.^27^ There are strong critiques on the use of narrow approaches that lack the involvement of priority populations, having co‐design approaches set before a project commences, and the process being managed by health‐care staff.^35^, ^36^

Undertaking a co‐design approach without the optimal conditions for inclusive involvement by all may not result in an equal partnership or improve health or care quality outcomes. ^37^, ^38^, ^39^ There is a risk of further marginalizing or adding burden to populations under‐represented and over‐researched with very little evidence that their involvement has improved the health system for them.^6^, ^13^, ^26^ Co‐design in health services is messy and complex, involving diverse actors with various abilities and statuses. Identifying diverse co‐design team members and bringing them together to enable and implement change requires an investment of resource before, during and after the process of co‐design.^40^, ^41^, ^42^, ^43^ Much of the work undertaken in well‐developed co‐design processes is invisible and based on values and relationships. This can involve additional time to enable the development of trust between participants or capture feedback and input outside of formal co‐design settings such as workshops, in particular for the public and patient partners.^19^ Co‐design work can increase the time and costs of involvement with no robust evidence of improved health outcomes.^16^, ^26^ Questions regarding the impact of undertaking a co‐design process must be considered.^15^, ^44^, ^45^ We argue that co‐design and associated terms must be considered over three stages, stressing the need to shift focus away on doing co‐design. Each has the potential to result in unintended consequences in the doing of co‐design if not considered, and we outline mitigation strategies as potential solutions.

## 3 PRE‐COMMENCEMENT STAGE: WHO DECIDES THAT CO‐DESIGN IS THE BEST APPROACH?

We recognize that there are now significant guidance, frameworks and literature on co‐designing once everyone is together.^46^, ^47^ There still is a substantial gap in the evidence on what needs to occur before participants enter into a co‐design process.^48^ Clarity is required on who decides what type of co‐design approach to take ^49^ The pre‐commencement stage should provide the space for exploring what other alternatives/approaches are available. Recent work found a lack of guidance on approaching this pre‐commencement stage, noting the phase where inequities of power concerning agenda setting were occurring.^13^ As a potential solution, the paper stressed the need to embed values‐based approaches such as respect, openness, reciprocity and flexibility from the pre‐commencement.^13^ Several ways these values could be used were outlined. For example, all partners planning co‐design work should use the values as a basis of developing an initial term of reference outlining everyone motivations on why they want to undertake co‐design. These should be constantly reflected upon by all parties. This requires time and being open to flexibility from all to engage repeated and transparent dialogue to achieve beneficial outcomes for all stakeholders. Being open to new inputs and differing modes of knowledge and ideas must be expected during this stage.

There is still a lack of clarity on how the key principles and practice of co‐design is implemented for populations who experience health and health‐care disparities, such as those from ethnic minority backgrounds.^19^, ^22^ A key task to undertake at the pre‐commencement stage should be an understanding of who should be involved. It is often the case that the ‘usual suspects’ are involved in co‐design work.^14^, ^35^, ^37^, ^49^, ^50^, ^51^ Understanding, building trust and engaging diverse public involvement are a key activity at the pre‐commencement stage.^11^, ^37^ This requires time and must be culturally appropriate.^43^ This may involve meetings between partners occurring in ‘spaces and places’ that are accessible and safe to them, often occurring in their own local communities.^14^

## 4 PROCESS OF INVOLVEMENT

Once co‐design commences, the main priority is to ensure that all participants are treated equally in the process.^18^, ^19^, ^52^ Power relations within these interactions can manifest easily often because of where the co‐design occurs.^32^, ^37^ The space and place where the co‐design occurs are often overlooked.^14^ Consider, for example, if the interactions are occurring in an acute hospital, external participants can often be unfamiliar with the environment. Often, they can be left to navigate this environment to make it to the ‘room’. This first interaction can often be overwhelming.^14^ Previous work has stressed the importance of developing a positive atmosphere that enables the contribution of all.^19^, ^47^, ^51^ One approach is to use community members as co‐chairs of the co‐design sessions and to provide them with adequate capacity building to enable them to amplify their voices.^19^ Having the co‐design sessions relaxed, informal jargon‐free that provided food and refreshments was also stressed.^19^ It is necessary to ensure that flexible and diverse financial (vouchers, cash or whichever is most appropriate for the community partners as alternatives) and administrative supports are provided to all participants. Managing and providing realistic and clear expectations regarding participant input into the co‐design process were highlighted as important enablers for meaningful involvement.^12^, ^14^, ^19^, ^36^, ^51^ Expecting and managing tensions must be anticipated, and it is important that everyone involved in the process can set the agenda and influence the process.^53^

## 5 AFTER CO‐DESIGNING, WHAT NEXT?

Ensuring ownership of the co‐design process must continue during the implementation stage. Often, people leave the co‐design space and think it is the responsibility of others to implement.^47^ Perhaps the most significant gap of co‐design is enabling a feedback loop for all who were involved in the process as the ideas are implemented and evaluated.^15^, ^37^ How can participants who were involved in the co‐design project have oversight and involvement in the implementation state? For example, Palmer et al note that people involved in co‐design, such as mental health service users, can often have a fractured experience in the health system. Their attitudes can change from being involved in co‐design, and they can be important champions to support organizational culture change.^2^ Often overlooked is defining and guiding the roles of implementation for those involved in the co‐design process—ensuring that all participants are invited to be involved is critical. The benefits of involving community groups and service users at this stage can have significant benefits as they can champion the change and communicate the change back to their communities.^13^

Co‐design is often so contextualized that we do not have enough evidence of how it can be scaled and spread for long‐term health system impact.^26^ We do know from the literature that a key mechanism for implementation of co‐design priorities is securing institutional commitment, sponsorship and leadership.^6^ An exemplar of this occurring focused on co‐designing a frailty pathway that stressed the importance of supporting collective leadership across the health system to support implementation.^19^ Education and training were critical to encourage professional commitment to the initiatives identified from the co‐design phase. Key to the success and future sustainability of many of the initiatives was identifying champions who could be a liaison between the different professions while negotiating disciplinary responsibilities and demonstrating strong interpersonal communication skills.^19^ Ensuring on‐going evaluations of co‐design initiatives is central to capture success where the focus is on capturing long‐term health system change.^2^, ^12^, ^14^, ^18^, ^26^, ^44^ The evidence on the impact of co‐design is short term, with scant evidence available of the long‐term changes occurring in the health system.^26^, ^32^ There needs to be a focus on long‐term reciprocal partners where co‐design initiatives are built on and developed to support transformation evidence. The key to this is helping people to step in and out of the co‐design process by enabling an on‐going learning system, mentoring and capacity building.^19^

Optimal conditions that can mitigate unintended consequences for co‐design arise when there is sufficient focus from the pre‐commencement to the implementation stages, with some core elements notable at each stage (table 1).

**Table 1:** Mitigation strategies for optimal co‐design

Pre‐Commencement	Mitigation Strategies
At the core of co‐design should be the involvement of diverse participants.	Time and resources are needed to understand who needs to be involved. At the core of this is building trust, and this work may occur in spaces and places that are inclusive and safe for partners.
Co‐design adds to the complexity of the work being undertaken. Is co‐design the right approach?	Central to good co‐design is allowing all stakeholders equal opportunities to influence the process. Using values‐based approaches can enable the development of an initial term of reference. This should capture everyone's motivations, noting the agreed outcome and should be monitored frequently.
Identify champions to support the implementation	The support and involvement of health‐care staff across the system are critical to enabling implementation. Senior leaders need to be included in this stage. Time and focus are needed at this stage on how to develop, agree, capture and evaluate long‐term impacts

Process of involvement	Mitigation Strategies
Enabling equal inclusive involvement.	All partners involved must be compensated for their time associated with participation (travel, subsistence, care release etc). Additional supports may need to be provided for some participants such as translation, follow‐up calls to answer any questions and accessibility supports.
Managing power relations	Tensions and conflicts can emerge during co‐design. To ensure democratic involvement, rotating lay co‐chair is recommended. This may require the provision of additional supports to amplify their voices.
Inclusive environments	Review and consider the space where the involvement will occur. Will it create an open atmosphere where everyone can feel included?
Managing expectations	Enable open communication that provides clear and realistic expectations for all. Review and refine via consensus the developed terms of reference

After co‐design	Mitigation Strategies
Ownership of the process	Ensure that role clarity for implementation is agreed upon by those who were involved in the process. Provide diverse opportunities to support oversight and champion implementation. This may need to involve further training and education sessions.
Update on implementation	Provide a feedback loop for all on implementation.
Capture impact	Review, reflect and share the impact. Ensure support for co‐design is provided by the health‐care organization to support any learnings and changes that emerge from the process.
Celebrate success and retain links	Ensure that time and adequate flexible resources are made available to celebrate success and achievements. Maintain on‐going relationships with partners by supporting on‐going learning and capacity building.

## 6 CO‐DESIGN MAY NOT BE SUITABLE FOR ALL INITIATIVES

To conclude, we recognize that co‐design is messy and may not be suitable for all initiatives.^14^, ^39^ Ensuring inclusive, equitable involvement is time‐consuming, and requires everyone involved to be flexible, patient and open to differing ideas. Much of the work involved is invisible. To do this well, it is clear from the literature and from our experiences of undertaking co‐design that the work requires on‐going reflective discussions and deliberative thinking to remove any power imbalances. However, without adequate resources, a focus on implementation and support from senior leaders, it is a tough ask to achieve. It often reinforces hierarchies and takes away the spirit of involvement that is central to co‐design.^14^, ^44^, ^49^ Senior leaders within health systems have a key role to play to support this, particularly at the pre‐commencement stage.^47^ The motivations for those working in the health system to scale up and ensure diversity of involvement of co‐design methodologies remain challenging.^3^, ^8^ Co‐design work must be responsive and open to mitigating the potential multiple unintended consequences from co‐design.

## Data Availability

Data sharing is not applicable to this article as no data sets were generated or analysed during the current study.
